# Clinical Consultation in the Workplace: Are There Implications for Response Attitudes?

**DOI:** 10.5334/pb.1346

**Published:** 2025-01-02

**Authors:** Bárbara Gonzalez, Rosa F. Novo, Maria João Afonso, Matilde Fernandes, Ana Vieira

**Affiliations:** 1 Lusófona University, HEI-Lab: Digital Human-Environment Interaction Labs, Portugal; 2 CICPSI (Research Centre in Psychological Science), Portugal; 3 Faculdade de Psicologia, Universidade de Lisboa, CICPSI (Research Centre in Psychological Science) –Alameda da Universidade, 1649-013 Lisbon, Portugal; 4 Faculdade de Psicologia, Universidade de Lisboa –Alameda da Universidade, 1649-013 Lisbon, Portugal; 5 DivPsi –PSP (Portuguese Police –Clinical Psychology Department), Unidade Especial de Polícia –Quinta das Águas Livres, 2605-197 Belas, Portugal

**Keywords:** MMPI-2-RF, clinical-organizational context, under-reporting, over-reporting, psychopathology

## Abstract

The clinical-organizational context (where clinical psychology services are provided in the individuals’ professional setting) has still been insufficiently approached in research, namely the influence it may have on the response attitudes of individuals undergoing psychological assessment. Our main goal is to find out if, when psychological assessment occurs in the workplace context, patients being assessed present specific response bias that may have implications for the clinical results and correlative decisions. Five hundred and ten adult participants grouped in two samples of ambulatory patients – Clinical-Organizational Sample (COS *n* = 238) and Clinical Sample (CS *n* = 272) – were assessed with the Minnesota Multiphasic Personality Inventory-2-RF validity and substantive scales. Under-reporting is five times more frequent in the COS, which presents Defensiveness (11%), and Desirability (5%). In the CS, under-reporting is residual and over-reporting is more prevalent than in the COS. Clinical record information of COS participants presenting under *vs*. over-reporting also reveal differences concerning their circumstances, and type of clinical conditions. Comparing participants with under-reporting in each sample, the COS had lower clinical profiles, and tended to present excessively low psychopathology and symptomology values, suggesting higher defensiveness. Finally, the fact that 33% of the COS present biased response attitudes (i.e., 15% presented under-reporting and 18% presented over-reporting) has implications for both clinical and career decision making processes. In conclusion, there are relevant differences in response attitude and psychopathology features between outpatients assessed in a traditional clinical setting and in a clinical-organizational one, suggesting the professional context of the patients may influence motivations to disclosure psychological symptoms and problems.

The clinical-organizational context is a very specific one and refers to the situation in which clinical psychology services are provided to workers in their professional setting (i.e., mental health care is delivered by the organization where the individuals work). Lowman (1979, as cited in Lowman, 2016) referred to the clinical-organizational context in the sense of a ‘clinical-organizational psychological model’ of organizational consultation. Clinical psychology inside working organizations is reflected on employee assistance programs, where individual employees and/or family members are assessed and provided either treatment, or referral to other providers. The workplace is already considered an important context for early identification and intervention on mental health problems, but there are still few studies focused on this context (e.g., Rothermund et al., 2016; Rothermund et al., 2017). To our knowledge, no study has yet approached the issue of response attitudes of patients being assessed in these services, which we believe is relevant to be explored, as it is possible that these response attitudes are different from the ones of patients assessed outside the workplace. The fact that an individual is undergoing psychological assessment in the organization where he works may enhance a response bias, which may impact psychological assessment results and have practical implications.

The context in which a psychological assessment takes place is very relevant, as it may affect the response attitudes to self-report psychological tests (Ziegler et al., 2012). These attitudes are crucial, as they may alter the results and undermine the very goal of psychological assessment, the capturing of real characteristics of the person being assessed, in whatever psychological variables are involved. Two opposite tendencies of response distortion are a threat to psychological assessment in any context, the under-reporting and the over-reporting of symptoms and undesirable personality characteristics.

Under-reporting pertains to a self-presentation of the individual being assessed that suggests a better level of functioning than would be indicated by a hypothetical objective assessment, and this term is preferred over terms as “faking good” or “positive malingering”, which connote an intentionality that cannot be inferred from test data alone (Ben-Porath & Tellegen, 2008/2011).

Two sub-types of under-reporting have been variously labeled, whose similar content is generally recognized: desirability, which pertains to an extremely positive self-image, through the exaggeration of desirable features and denial of any kind of flaw; and defensiveness, which occurs when the individual denies psychological problems and symptoms in order to appear psychologically adjusted (Rogers, 2018). These dimensions have also been labeled as positive impression management and self-deception, respectively (e.g., Paulhus, 1984, cited in Bagby & Marshal, 2004). The two under-reporting sub-types, desirability and defensiveness, are not mutually exclusive and people may present both (Rogers, 2018). The tendency to conceal or minimize psychological symptoms and engage in a positive impression management has been shown to be higher in high stakes contexts, where assessment results have relevant practical implication for individuals being assessed (Picard et al., 2023; Rogers, 2018).

Over-reporting is defined as a self-presentation portraying of a more extreme degree of dysfunction than would be indicated by a hypothetical objective assessment of the individual. This term is preferred over expressions that imply inferences of intentionality, as ‘faking bad’ or ‘malingering’ (Ben-Porath & Tellegen, 2008/2011). Actually, in mental health contexts, individuals may present over-reporting for other reasons than intentional simulation, and this should be contextualized in order to avoid the negative connotation that label implies (Merckelbach et al., 2019). The response attitudes of under and of over-reporting are multidetermined and influenced by context features, and by personal characteristics (Rogers, 2018; Ziegler et al., 2012).

The Minnesota Multiphasic Personality Inventory 2-RF (MMPI-2-RF (Ben-Porath & Tellegen, 2008/2011) is a self-report inventory assessing personality and psychopathology. This updated version, and its predecessor, the MMPI-2, are among the most extensively used worldwide psychometric instruments, for psychological assessment in several contexts. It is a reference test, for its different types of validity scales, assessing both under and over-reporting, and for the broad range of personality and psychopathology scales. Research has shown that it is able to discriminate participants presenting and not presenting an under-reporting attitude (e.g., Crighton et al., 2017; Jiménez-Gómez et al., 2013; Picard et al., 2023), and an over-reporting attitude (Ingram & Ternes, 2016; Sharf et al., 2017), as well as the impact of these validity scales’ scores on the substantive clinical scales. A recent systematic review and preliminary meta-analysis has shown that translated versions of the instrument are also effective in detecting both tendencies of response distortion (Bopp et al., 2022). Pertaining to the two dimensions of under-reporting, the L-r scale assesses impression management and the K-r scale assesses self-deception (Bagby & Marshall, 2004).

Although this instrument has been extensively used in different contexts (e.g., parental custody, professional selection, disability claiming), to our best knowledge, it has not been used to identify possible features that would characterize the response attitudes and concomitant clinical aspects in individuals assessed with clinical purposes inside a clinical-organizational context. The organizational context is one of the contexts in which positive impression management, including underreport of undesirable personality characteristics or psychological symptoms, is expected, due to a fear of a negative impact in getting or maintaining a job (Picard et al., 2023). A meta-analysis has found that individuals applying for a job distort their scores on measures of personality to portray themselves in a positive manner (Birkeland et al., 2006). In this vein, research has shown that employees are afraid to disclosure mental health problems in the workplace (e.g., Brohan et al., 2012; Stratton et al., 2018), and a study in 35 countries showed that people with major depressive disorder very frequently reported discrimination in the work setting (Brouwers et al., 2016). Therefore, we question if being psychologically assed and followed in the organizational context, although with a clinical purpose and not for selection or fitness for duty, may still affect their response attitude.

This study aims at verifying if individuals with clinical complaint assessed in the workplace context (with clinical purposes and following their request for psychological help) present a pattern of response bias that is different from the one of patients being assessed in a traditional clinical context. We hypothesize that the former will present higher levels of under-reporting, mainly the tendency to deny psychological problems and relational difficulties, due to implications this might have in the work context. The research strategy consists of comparing two samples of ambulatory patients with clinical complaints, all of them involved in professional activity, assessed in two different contexts.

To our best knowledge, this research is the first to explore response attitudes and concomitant clinical features within the clinical-organizational field, concerning clinical purposes (and neither selection nor fitness for duty evaluations), in comparison with a traditional clinical context.

## Method

### Participants

The study included 510 adult participants from a database of psychological assessment with the MMPI-2-RF, which is managed by researchers from a research centre of a Portuguese university. which collect data in collaboration with several national institutions.

These participants were grouped in two samples. The first sample (Clinical-Organizational Sample or COS) included 238 patients presenting a clinical complaint and being assessed in the clinical psychology services provided by their working organizations, free of cost. The participants were transport companies’ employees, namely, air and railroad, and security forces employees. These clinical psychology services are ambulatory clinical counseling and psychotherapy services, not emergency ones. The second sample (Clinical Sample or CS) included 272 patients presenting a clinical complaint and being assessed by clinical psychologists in public mental health services, free of cost and ambulatory, not emergency services. The inclusion criterion was having professional activity, meaning all participants were professionally active. Both samples were, then, composed of ambulatory patients who searched for psychological help, all of them professionally active, currently working or in sick leave. All the patients, in both samples, had been referred to psychological assessment for diagnostic purposes, after a clinical interview with a psychologist/psychiatrist. Sociodemographic and clinical characteristics of the samples are presented in Table 1.

**Table 1:** Sociodemographic Variables by Sample and Statistics.


	CLINICAL-ORGANIZATIONAL SAMPLE (COS) (*N* = 238)		CLINICAL SAMPLE (CS) (*N* = 272)		STATISTICS

	*n (%)*	*M (SD)*	*n (%)*	*M (SD)*	

Gender					χ^2^(2) = 12.34, *p <* .001

Female	33 (14)		72 (26)		

Male	205 (86)		200 (74)		

Work Situation					

Working	211 (89)		214 (79)		χ^2^(2) = 9.10, *p <* .01

Sick Leave	27 (11)		58 (21)		

Major Clinical Condition					

Anxiety/Mood	157 (66)		185 (68)		χ^2^(3) = 2.02, *p =* .567

Behavior	43 (18)		44 (16)		

Substances	14 (6)		22 (8)		

Others/Not identified	24 (10)		21 (8)		

Self-identified problems					

Professional	84 (35)		94 (35)		χ^2^(3) = 1.05, *p =* .789

Relational/Family	51 (21)		60 (22)		

Physical health	65 (27)		82 (30)		

Others/Not Identified	38 (16)		36 (13)		

Clinical History					

Psychological/Psychiatric Intervention	60 (25)		66 (24)		χ^2^(1) = .061, *p =* .805

Psychotropic Medication	33 (14)		38 (14)		

Age		42.87 (9.18)		41.51 (11.61)	*F*(1, 508) = 2.11, *p =* .147

School Years		11.63 (2.15)		11.69 (3.17)	*F*(1, 508) = 0.07, *p* = .791


The samples sociodemographic characteristics reveal no significant differences according to age, education level, type of conditions identified by the psychologist performing the assessment, types of problems identified by the patients as leading to their psychological complaint, and clinical history. There are significant differences in gender and in current working situation.

## Instruments

### Minnesota Multiphasic Personality Inventory–2–Restructured Form

The national version of the inventory (Ben-Porath & Tellegen, 2008/2011; Novo et al., 2023) was used. The instrument includes different sets of scales that were used in this study: Validity scales (VS); Higher-Order scales (H-O); Restructured Clinical scales (RC); Specific Problems scales (Somatic/Cognitive – SOM/COG; Internalizing – INT; Externalizing – EXT; and Interpersonal – INTER); and Personality Psychopathology Five scales (PSY-5). Specifically, two validity scales assess under-reporting: Uncommon Virtues (L-r), which identifies the denial of common shortcomings, and Adjustment Validity (K-r), which identifies the tendency to claim unrealistically positive psychological adjustment. Five validity scales assess over-reporting: Infrequent Responses (F-r), which identifies unusual responses in general population; Infrequent Psychopathology Responses (Fp-r), which identifies unusual responses in psychiatric groups; Infrequent Somatic Responses (Fs), which identifies infrequent responses in medical patients populations; Symptom Validity (FBS-r), which identifies somatic and cognitive complaints associated at high levels of over-reporting; and Response Bias Scale (RBS), which identifies exaggerated memory complaints.

The results are converted into normalized T-scores for the Validity scales, and uniform T-scores for all remaining substantive scales. In general terms, scores > T64 are considered clinically significant values for the substantive scales.

#### Psychosocial Data Record

This self-report record contains data pertaining to the current clinical situation (psychological complaints and symptoms, types of problems identified by the patients as leading to their psychological complaint) and general data on the family household, schooling, professional activity, and health.

#### Clinical Information Form

Types of clinical conditions in the present, and clinical history data (e.g., clinical psychology/psychiatric consultation/treatment, psychiatric hospitalization, and/or psychotropic medication in the past) are filled by the psychiatrist or the clinical psychologist who requested the psychological assessment.

### Procedure

The COS protocols were gathered through the collaboration of psychologists working in organizational clinical services. The CS cases were extracted from a broad database, from which participants with demographic and clinical features similar to the ones of the COS were extracted (i.e., age, schooling years, type of clinical problems, and evidence or not of psychological or pharmacological intervention in the past).

In both samples, the MMPI-2-RF was administered individually by clinical psychologists within the scope of clinical psychological assessment, in accordance with the test standardized guidelines and scientific research norms. After this administration, the participants filled the Clinical and Psychosocial Data Record. Participants signed an informed consent and privacy was in conformity with the international principles for psychological research. The research was approved by the Ethic Committee of the (Faculty of Psychology of University of Lisbon).

The protocols with response omissions (Cannot Say) > 10 or with levels of response inconsistency (Variable Response Inconsistency – VRIN-r or True Response Inconsistency – TRIN-r) > T80 were removed from the database.

### Data analysis

Descriptive statistics to characterize the Clinical-Organizational and the Clinical Samples according to their participants gender, age, school years, types of clinical conditions identified by their psychologist, types of problems identified by the patients as leading to their psychological complaint, and clinical history were performed. Chi-squared tests were performed to assess differences between the samples defined by gender, work situation (currently working *vs*. on sick leave), types of clinical conditions identified by the psychologist, types of problems identified by the patients, and clinical history. Univariate analyses of variance (ANOVA) were performed to assess differences in age and school years.

Descriptive statistics (mean, standard deviation and confidence intervals at 95%) were used to compare the two samples in the validity and substantive scales’ scores. In accordance with standard norms, T-scores (*M* = 50, *SD* = 10) are used (Ben-Porath & Tellegen, 2008/2011).

A multivariate analysis of covariance (MANCOVA) was conducted to examine potential differences in MMPI-2-RF Validity, Higher-Order, and Restructured Clinical scales results between samples, while controlling for differences in gender and in work situation.

In order to analyze different types of response attitudes, we categorized the participants based on their scores on the MMPI-2-RF validity scales.

As the results of each validity scale, when individually analyzed, may have several interpretations (Ben-Porath & Tellegen, 2008/2011), we chose to eliminate protocols with high response inconsistency (see Procedure), and associate all scales of each response bias type (under-reporting, and over-reporting). Therefore, we intended to highlight a more specific interpretation and identify different levels of potential response bias.

The cut-off points follow the recommended values for each validity scale (Ben-Porath & Tellegen, 2008/2011) as likely to present ‘*Without Evidence’* or ‘*Possible’ Under or Over-Reporting*. In the case of ‘*Possible’ Under or Over-Reporting*, two categories were considered – Global Attitude and Specific Attitude – both for under-reporting and over-reporting. Global Attitude applies when all Under or Over indicators are present; Specific Attitude applies when only a few Under or Over indicators are present. Based on these criteria, the participants of each sample were categorized in the following categories:

- *Without Evidence of Under-Reporting:* L-r < T65 & K-r < T60
- *Possible Under-Reporting*
  - *Global Attitude*: L-r ≥ T65 & K-r ≥ T60);
  - *Specific Social Desirability:* L-r ≥ T65 & K-r < T60;
  - *Specific Defensiveness:* L-r < T65 & K-r ≥ T60
- *Without Evidence of Over-Reporting:* [(F-r < T80 & Fp-r < T70 & Fs < T80 & FBS-r < T80 & RBS < T80)]
- *Possible Over-Reporting*
  - *Global Attitude*: (F-r ≥ T80 | Fp-r ≥ T70) & (Fs ≥ T80 | FBS-r ≥ T80 | RBS ≥ T80);
  - *Specific Severe/Rare Complaints:* (F-r ≥ T80 & Fp-r ≥ T70) & (Fs < T80 & FBS-r < T80 & RBS < T80);
  - *Specific Somatic/Cognitive Complaints:* [(F-r < T80 & Fp-r < T70 & (Fs ≥ T80 & FBS-r ≥ T80)] | [(Fs ≥ T80 & RBS ≥ 80)] | [(FBS-r ≥ T80 & RBS ≥ T80)].

Throughout the paper, these categories will be more briefly designated as With or Without Under- or Over-Reporting, and Global or Specific attitude.

Frequencies for each category in each sample were calculated and Odd ratios (OR) were used to assess the likelihood of occurrence of under-reporting and over-reporting in the clinical-organizational context in comparison to the clinical context.

To examine potential differences in the Higher-Order and in the Restructured Clinical scales scores between the participants of both samples, with Under-Reporting, and with Over-Reporting attitudes, means, standard deviations, and multivariate analysis of variance (MANOVA) were performed.

Focusing only on the COS, we compared the participants with Under-Reporting, with Over-Reporting, and Without Under or Over-Reporting in the Higher-Order, Restructured Clinical, Specific Problems, and Personality Psychopathology scales scores, using descriptive statistics (mean, standard deviation and 95% confidence intervals).

Finally, we analyzed the clinical record information of the participants of the Clinical-Organizational Sample, classified as ‘with evidence of Under-Reporting’, and ‘with evidence of Over-Reporting’, in order to identify potential differences in four categories: self-identification of problems leading to the clinical complaint, sick leave due to physical or mental health problems, types of clinical conditions identified by the psychologist performing the assessment, and self-reported history of clinical psychology/psychiatric consultation and/or psychotropic medication.

## Results

The MANCOVA revealed a significant difference between the two samples on the combined dependent variables: Validity scales *F*(7, 500) = 10.14, *p* < .001, Wilks’ Λ = .88, partial η^2^ = .12; Higher-Order scales *F*(3, 505) = 19.02, *p* < .001, Wilks’ Λ = .90, partial η^2^ = .10; and Clinical Restructured scales *F*(9, 499) = 9.12, *p* < .001, Wilks’ Λ = .86, partial η^2^ = .14.

The descriptive statistics for each scale are presented in Table 2.

**Table 2:** Descriptive Statistic and Cohen’s d for the Validity, Higher-Order, and Restructured Clinical Scales in the Clinical-Organizational and Clinical Samples.


	CLINICAL ORGANIZATIONAL SAMPLE (COS) (*N* = 238)				CLINICAL SAMPLE (CS) (*N*= 272)				COHEN’S D (COS *VS* CS)
		
	*M*	*SD*	*95% CI*		*M*	*SD*	*95% CI*		
	
			*LL*	*UL*			*LL*	*UL*	

Validity Scales									

F-r	56.64	15.40	54.68	58.61	65.64	14.70	63.89	67.40	–0.60

Fp-r	52.12	11.82	50.61	53.63	60.10	13.29	58.52	61.69	–0.63

Fs	54.18	12.93	52.53	55.84	59.74	13.53	58.12	61.35	–0.42

FBS-r	54.53	13.05	52.86	56.19	58.53	12.33	57.05	60.00	–0.32

RBS	55.66	14.61	53.79	57.52	60.80	13.80	59.15	62.45	–0.36

L-r	49.71	9.82	48.46	50.96	45.96	9.60	44.81	47.11	0.40

K-r	48.10	9.98	46.82	49.37	41.88	7.62	40.97	42.79	0.71

Higher-Order Scales									

EID	55.21	12.95	53.55	56.86	62.71	11.31	61.36	64.06	–0.62

THD	54.22	11.68	52.73	55.71	60.81	12.77	59.28	62.33	–0.46

BXD	53.47	10.39	52.14	54.80	57.80	12.06	56.36	59.24	–0.39

Restructured Clinical Scales									

RCd	55.40	12.74	53.78	57.03	63.29	10.59	62.03	64.56	–0.68

RC1	52.88	11.01	51.47	54.28	57.99	9.18	56.89	59.09	–0.51

RC2	54.88	13.30	53.18	56.58	60.43	12.08	58.99	61.87	–0.44

RC3	51.16	9.72	49.91	52.40	55.11	10.16	53.89	56.32	–0.40

RC4	54.35	11.48	52.89	55.82	61.73	12.56	60.23	63.23	–0.61

RC6	56.31	12.31	54.74	57.88	61.20	12.95	59.65	62.74	–0.40

RC7	53.53	11.68	52.03	55.02	59.64	10.13	58.43	60.85	–0.56

RC8	53.00	11.39	51.54	54.45	60.51	12.32	59.04	61.98	–0.63

RC9	51.50	9.49	50.29	52.71	54.67	9.90	53.49	55.85	–0.36


*Note. CI* = confidence interval; *LL* = lower limit; *UL* = upper limit. F-r = Infrequent Responses; Fp-r = Infrequent Psychopathology Responses; Fs = Infrequent Somatic Responses; FBS-r = Symptom Validity; RBS = Response Bias Scale; L-r = Uncommon Virtues; K-r = Adjustment Validity; EID = Emotional/Internalizing Dysfunction; THD = Thought Dysfunction; BXD = Behavioral/Externalizing Dysfunction; RCd = Demoralization; RC1 = Somatic Complaints; RC2 = Low Positive Emotions; RC3 = Cynicism; RC4 = Antisocial Behavior; RC6 = Ideas of Persecution; RC7 = Dysfunctional Negative Emotions; RC8 = Aberrant Experiences; RC9 = Hypomanic Activation.

There were significant differences between the two samples in all Validity scales. The COS presented lower scores in all over-reporting five scales and higher scores in the two under-reporting scales. In the Higher-Order scales, the COS reported less psychological difficulties at the different levels, in THD (i.e., problems associated with thinking disorder), in EID (i.e., problems associated with mood and affect), and in BXD (i.e., problems related with under-controlled behavior. In the Restructured Clinical scales, the differences were significant in all scales, with COS presenting lower values of psychopathology.

In regard to the response attitude of the participants, different types of Under- and Over-Reporting were identified in each sample (see Table 3).

**Table 3:** Types of Response Attitude in the Clinical-Organizational and the Clinical Samples.


	CLINICAL-ORGANIZATIONAL SAMPLE (COS) (*N*= 238)		CLINICALSAMPLE (CS) (*N* = 272)		COS : CS	
		
	*F*	%	*F*	%	*OR*	95% CI

**Under-reporting**						

Without	203	85	263	97		

With	35	15	9	3	5.04*	[2.37, 10.72]

Global	(5)		(0)			

Social Desirability	(8)		(6)			

Defensiveness	(22)		(3)			

**Over-reporting**						

Without	194	82	180	66		

With	44	18	92	34	0.44*	[0.29, 0.67]

Global	(20)		(35)			

Severe/Rare Complaints	(3)		(11)			

Somatic/Cognitive Complaints	(3)		(5)			

Not considered in any specific category^§^	(18)		(41)			

*Without Under and/or Over-Reporting*	159	67	171	63		


*Note*. ^§^ Participants that present only one type of Severe/Rare Complaint or one type of Somatic/Cognitive Complaint do not fit into any of these specific categories. * *p* < .001.

In both samples, the number of individuals who showed neither Under- nor Over-Reporting response attitudes is relatively close: 67% in the COS and 63% the CS. However, the response attitudes of both samples were quite different. In the ‘traditional’ CS, under-reporting was residual and over-reporting was mainly of the Global type (i.e., with individuals presenting severe/rare and somatic/cognitive complaints, simultaneously). In the COS, under- and over-reporting were both present at similar levels, with 15% and 18% incidence, respectively. Within under-reporting, the sub-type Defensiveness prevailed, and within over-reporting, the Global type prevailed. The presence of both under- and over-reporting was not identified in any participant of both samples.

Table 3 depicts Odd Ratio estimates significant (with 95% confidence intervals, not including 1), being greater than 1 in under-reporting, suggesting it was more likely in the clinical-organizational context, and being smaller than 1 in over-reporting, suggesting it was less likely in this context than in the “traditional” clinical one.

Considering the groups of participants presenting under-reporting or over-reporting, in each sample, we analyzed each scale mean score and the effect size of the differences between the samples (see Table 4).

**Table 4:** Descriptive Statistic and ANOVA for the Substantive Scales in the Clinical-Organizational and Clinical Samples.


SCALES	CLINICAL-ORGANIZATIONAL SAMPLE (COS) (*N* = 238)				CLINICAL SAMPLE (CS) (*N* = 272)				OVER-REPORTING COS (*n* = 44) CS (*n* = 92) ANOVA		UNDER-REPORTING COS (*n* = 35) CS (*n* = 9) ANOVA	
	
	OVER-REPORTING (*n* = 44)		UNDER-REPORTING (*n* = 35)		OVER-REPORTING (*n* = 92)		UNDER-REPORTING (*n* = 9)					

	*M*	*SD*	*M*	*SD*	*M*	*SD*	*M*	*SD*	*F*	*η_P_^2^*	*F*	*η_P_^2^*

Higher-Order Scales												

EID	71.14	10.69	42.23	6.67	69.83	10.72	48.56	4.75	.45	‒	7.11*	0.15

THD	69.34	12.31	47.09	7.54	71.43	11.66	52.78	9.50	.93	‒	3.67	‒

BXD	58.45	12.34	46.60	7.00	60.54	12.23	56.44	10.83	.86	‒	11.19**	0.21

Restructured Clinical Scales												

RCd	71.75	9.83	42.74	5.29	70.47	9.16	50.67	4.56	.56	‒	16.97***	0.29

RC1	67.82	8.95	43.71	6.04	65.72	8.57	52.67	6.00	1.74	‒	15.76***	0.27

RC2	67.45	14.25	44.20	8.25	64.78	12.19	49.33	4.12	1.28	‒	3.23	‒

RC3	59.52	10.30	41.09	5.32	60.30	9.38	51.44	13.38	.19	‒	13.47**	0.24

RC4	61.52	11.78	46.23	6.95	65.29	12.21	57.89	8.98	2.91	‒	17.86***	0.30

RC6	70.25	12.95	47.11	9.00	70.60	12.76	55.22	10.77	.02	‒	5.37*	0.11

RC7	68.48	9.27	41.71	6.62	67.07	10.11	50.89	4.05	.06	‒	15.62***	0.27

RC8	68.14	11.11	45.31	6.96	70.43	10.94	50.67	6.38	1.30	‒	4.64*	0.09

RC9	56.02	11.21	43.80	6.54	58.36	10.15	47.67	4.69	1.47	‒	2.76	‒


*Note*. EID = Emotional/Internalizing Dysfunction; THD = Thought Dysfunction; BXD = Behavioral/Externalizing Dysfunction; RCd = Demoralization; RC1 = Somatic Complaints; RC2 = Low Positive Emotions; RC3 = Cynicism; RC4 = Antisocial Behavior; RC6 = Ideas of Persecution; RC7 = Dysfunctional Negative Emotions; RC8 = Aberrant Experiences; RC9 = Hypomanic Activation. *p < .05. **p < .01. ***p < .001.

Pertaining to **Under-Reporting**, the COS had very low mean values in the substantive scales, as all of their scores were beyond T48, while in the Clinical Sample no scale was beyond that value (all means above T56). The MANOVA showed significant differences between the two samples in the composites of both Higher-Order scales (Wilk’s Λ = .68; *F*(3,40) = 6.20; *p* = .001; η^2^par = .32) and Restructured Clinical scales (Wilk’s Λ = .42; *F*(9,34) = 5.17; *p* < .001; η^2^par = .58). There were significant differences in nine of the 12 scales, and with high effect size > .20 in six of them. In the COS, four scales stand out, showing significant differences from the CS and with mean values < T43, highlighting the tendency to an almost extreme tendency to deny any kind of problems or difficulties: EID (Problems associated with mood and affect), RCd (General unhappiness and dissatisfaction), RC3 (Non-self-referential beliefs that others are bad and not to be trusted), RC7 (Maladaptive anxiety, anger, and irritability).

Pertaining to **Over-Reporting**, the MANOVA showed no significant differences between the two samples in the composites of both H-O (Wilk’s Λ = .99; *F*(3,132) = .59; *p* = .620; η^2^par = .01) and RC (Wilk’s Λ = .95; *F*(9,126) = .79; *p* = .624; η^2^par = .05) scales.

Analyzing the effect of response attitudes on the psychopathology scales, in the COS, specifically, we compared the mean scores in all sets of substantive scales (H-O, RC, Specific Problems, and Personality Psychopathology Five) (see Table 5) of participants presenting under or over-reporting with participants that did not present any of the biased response attitudes. This last group was considered the reference group to identify the areas where under and over-reporting are manifested the most.

**Table 5:** Descriptive Statistics for the Substantive Scales in the Clinical-Organizational Subsamples.


	CLINICAL-ORGANIZATIONAL SUBSAMPLES											

	UNDER-REPORTING (*n* = 35)				OVER-REPORTING (*n* = 44)				WITHOUT UNDER- OR OVER-REPORTING (*n* = 159)			
		
	*M*	*SD*	*95% CI*		*M*	*SD*	*95% CI*		*M*	*SD*	*95% CI*	
		
			*LL*	*UL*			*LL*	*UL*			*LL*	*UL*

Higher-Order Scales												

EID	42.23	6.67	39.94	44.52	71.14	10.69	67.89	74.39	53.65	10.02	52.08	55.22

THD	47.09	7.54	44.49	49.68	69.34	12.31	65.60	73.08	51.60	8.33	50.30	52.91

BXD	46.60	7.00	44.20	49.00	58.45	12.34	54.70	62.21	53.60	9.62	52.10	55.11

Restructured Clinical Scales												

RCd	42.74	5.29	40.93	44.56	71.75	9.83	68.76	74.74	53.67	9.90	52.12	55.22

RC1	43.71	6.04	41.64	45.79	67.82	8.95	65.10	70.54	50.76	8.17	49.48	52.04

RC2	44.20	8.25	41.37	47.03	67.45	14.25	63.12	71.79	53.75	11.18	52.00	55.50

RC3	41.09	5.32	39.26	42.91	59.52	10.30	56.39	62.66	51.06	8.04	49.80	52.32

RC4	46.23	6.95	43.84	48.62	61.52	11.78	57.94	65.10	54.16	10.98	52.44	55.88

RC6	47.11	9.00	44.02	50.21	70.25	12.95	66.31	74.19	54.48	9.39	53.01	55.95

RC7	41.71	6.62	39.44	43.99	68.48	9.27	65.66	71.30	51.99	8.73	50.62	53.35

RC8	45.31	6.96	42.92	47.71	68.14	11.11	64.76	71.51	50.50	8.20	49.21	51.78

RC9	43.80	6.54	41.55	46.05	56.02	11.21	52.61	59.43	51.94	8.53	50.61	53.28

Specific Problems: Somatic Scales												

MLS	42.91	5.95	40.87	44.96	68.41	8.69	65.77	71.05	55.56	11.26	53.80	57.32

GIC	45.00	0.00	45.00	45.00	63.86	14.30	59.52	68.21	51.18	10.89	49.47	52.88

HPC	43.89	5.58	41.97	45.80	66.55	9.04	63.80	69.29	49.88	9.22	48.44	51.32

NUC	44.94	6.76	42.62	47.26	66.66	9.24	63.85	69.47	50.32	9.25	48.87	51.77

COG	43.83	6.33	41.65	46.00	70.20	11.51	66.71	73.70	52.26	11.19	50.51	54.02

Specific Problems: Internalizing Scales												

SUI	49.17	7.33	46.65	51.69	76.66	22.14	69.93	83.39	54.67	14.76	52.35	56.98

HPL	43.46	7.33	40.94	45.97	64.55	11.34	61.10	67.99	50.87	9.75	49.34	52.40

SFD	44.74	4.65	43.15	46.34	65.25	10.48	62.06	68.44	51.54	11.15	49.80	53.29

NFC	43.54	8.26	40.71	46.38	62.75	10.77	59.48	66.02	51.21	9.76	49.68	52.74

STW	43.77	7.74	41.11	46.43	62.52	10.14	59.44	65.61	52.09	9.60	50.59	53.60

AXY	45.14	7.12	42.70	47.59	70.27	11.41	66.80	73.74	51.64	9.47	50.16	53.12

ANP	42.94	4.41	41.43	44.46	66.39	10.76	63.12	69.66	51.24	10.48	49.60	52.88

BRF	45.60	6.90	43.23	47.97	59.25	9.82	56.27	62.23	49.22	7.78	48.00	50.44

MSF	46.06	7.96	43.32	48.79	50.34	9.94	47.32	53.36	47.53	8.03	46.27	48.79

Specific Problems: Externalizing Scales												

JCP	48.37	8.67	45.39	51.35	55.27	12.57	51.45	59.09	52.67	10.68	50.99	54.34

SUB	47.74	7.69	45.10	50.39	59.93	16.95	54.78	65.08	52.48	12.47	50.53	54.43

AGG	43.26	5.88	41.24	45.28	65.98	14.03	61.71	70.24	54.38	10.80	52.69	56.08

ACT	46.14	9.38	42.92	49.36	54.70	9.58	51.79	57.62	50.36	8.29	49.07	51.66

Specific Problems: Interpersonal Scales												

FML	43.37	5.93	41.33	45.41	61.82	11.63	58.28	65.35	50.84	9.51	49.35	52.33

IPP	47.46	8.12	44.67	50.25	56.59	14.36	52.22	60.96	49.57	9.74	48.04	51.09

SAV	47.66	8.58	44.71	50.60	66.02	13.15	62.02	70.02	53.57	11.05	51.84	55.30

SHY	44.40	6.52	42.16	46.64	61.77	11.06	58.41	65.14	51.42	10.32	49.81	53.04

DSF	47.69	8.71	44.69	50.68	72.59	16.49	67.58	77.61	52.36	12.10	50.46	54.25

Personality Psychopathology Five (PSY-5) Scales												

AGGR-r	50.63	7.69	47.99	53.27	51.91	16.02	47.04	56.78	52.55	10.53	50.90	54.20

PSYC-r	46.14	7.18	43.68	48.61	68.48	10.84	65.18	71.77	49.97	8.21	48.69	51.26

DISC-r	50.97	6.79	48.64	53.30	54.23	11.36	50.77	57.68	53.30	8.43	51.98	54.62

NEGE-r	42.80	6.64	40.52	45.08	67.50	10.99	64.16	70.84	51.92	8.87	50.54	53.31

INTR-r	48.29	8.73	45.29	51.29	66.75	14.05	62.48	71.02	53.91	11.18	52.16	55.66


*Note. CI* = confidence interval; *LL* = lower limit; *UL* = upper limit. EID = Emotional/Internalizing Dysfunction; THD = Thought Dysfunction; BXD = Behavioral/Externalizing Dysfunction; RCd = Demoralization; RC1 = Somatic Complaints; RC2 = Low Positive Emotions; RC3 = Cynicism; RC4 = Antisocial Behavior; RC6 = Ideas of Persecution; RC7 = Dysfunctional Negative Emotions; RC8 = Aberrant Experiences; RC9 = Hypomanic Activation; MLS = Malaise; GIC = Gastrointestinal Complaints; HPC = Head Pain Complaints; NUC = Neurological Complaints; COG = Cognitive Complaints; SUI = Suicidal/Death Ideation; HLP = Helplessness/Hopelessness; SFD = Self-Doubt; NFC = Inefficacy; STW = Stress/Worry; AXY = Anxiety; ANP = Anger Proneness; BRF = Behavior -Restricting Fears; MSF = Multiple Specific Fears; JCP = Juvenile Conduct Problems; SUB = Substance Abuse; AGG = Aggression; ACT = Activation; FML = Family Problems; IPP = Interpersonal Passivity; SAV = Social Avoidance; DSF = Disaffiliativeness; AGGR-r = Aggressiveness-Revised; PSYC-r = Psychoticism-Revised; DISC-r = Disconstraint-Revised; NEGE-r = Negative Emotionality/Neuroticism-Revised; INTR-r = Introversion/Low Positive Emotionality-Revised.

Considering the confidence interval at 95%, participants with under-reporting had lower mean scores than the reference group in all H-O, RC and Somatic sales, all Internalizing scales (except for MSF), all Interpersonal scales (except for IPP), and all PSY-5 (except for AGGR-r and DISC-r). In the Externalizing scales, AGGR-r was the only scale where no differences were found.

Pertaining to over-reporting, this group had higher mean scores in the H-O scales (except for BXD), all RC scales (except for RC9), all Somatic scales, all Internalizing scales (except for MSF), and all Interpersonal scales; in the Externalizing scales, AGGR-r was the only scale in which the over-reporting group had significantly higher scores. In the Personality Psychopathology scales PSYC-r and NEGE-r, this group had significantly higher mean scores than the reference group, while in the AGGR-r and DISC-r, there were no significant differences.

**Figure 1:**
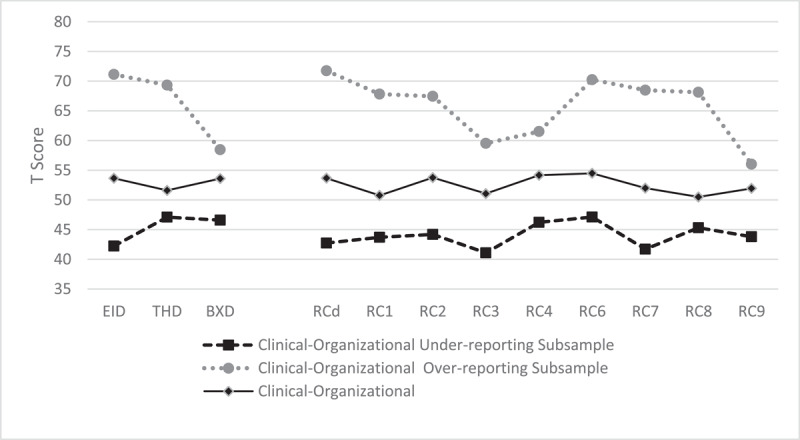
Mean Profiles of Clinical-Organizational Subsamples on the Higher-Order and Restructured Clinical Scales. *Note*. Clinical-Organizational Under-Reporting Subsample: *n* = 35; Clinical-Organizational Over-Reporting Subsample: *n* = 44; Clinical-Organizational Without Under- or Over-Reporting Subsample: *n* = 159. EID = Emotional/Internalizing Dysfunction; THD = Thought Dysfunction; BXD = Behavioral/Externalizing Dysfunction; RCd = Demoralization; RC1 = Somatic Complaints; RC2 = Low Positive Emotions; RC3 = Cynicism; RC4 = Antisocial Behavior; RC6 = Ideas of Persecution; RC7 = Dysfunctional Negative Emotions; RC8 = Aberrant Experiences; RC9 = Hypomanic Activation.

**Figure 2:**
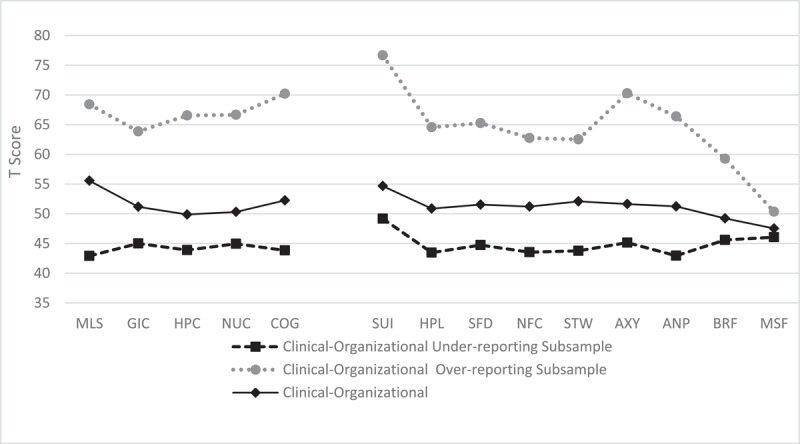
Mean Profiles of Clinical-Organizational Subsamples on the Specific Problems: Somatic and Internalizing Scales. *Note*. Clinical-Organizational Under-Reporting Subsample: *n* = 35; Clinical-Organizational Over-Reporting Subsample: *n* = 44; Clinical-Organizational Without Under- or Over-Reporting Subsample: *n* = 159. MLS = Malaise; GIC = Gastrointestinal Complaints; HPC = Head Pain Complaints; NUC = Neurological Complaints; COG = Cognitive Complaints; SUI = Suicidal/Death Ideation; HLP = Helplessness/Hopelessness; SFD = Self-Doubt; NFC = Inefficacy; STW = Stress/Worry; AXY = Anxiety; ANP = Anger Proneness; BRF = Behavior -Restricting Fears; MSF = Multiple Specific Fears.

**Figure 3:**
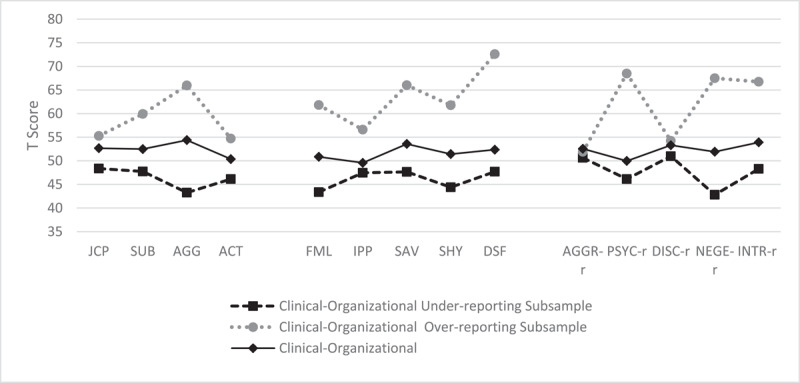
Mean Profiles of Clinical-Organizational Subsamples on the Specific Problems: Externalizing, Interpersonal and Personality Psychopathology Scales. *Note*. Clinical-Organizational Under-Reporting Subsample: *n* = 35; Clinical-Organizational Over-Reporting Subsample: *n* = 44; Clinical-Organizational Without Under- or Over-Reporting Subsample: *n* = 159. JCP = Juvenile Conduct Problems; SUB = Substance Abuse; AGG = Aggression; ACT = Activation; FML = Family Problems; IPP = Interpersonal Passivity; SAV = Social Avoidance; DSF = Disaffiliativeness; AGGR-r = Aggressiveness-Revised; PSYC-r = Psychoticism-Revised; DISC-r = Disconstraint-Revised; NEGE-r = Negative Emotionality/Neuroticism-Revised; INTR-r = Introversion/Low Positive Emotionality-Revised.

In Figures 1, 2, and 3, the mean profiles of each group in the different sets of scales are shown.

Finally, to explore clinical information associated with the psychological consultation request of individuals that present biased response attitudes, we compared the two subgroups of the Clinical-Organizational Sample, with under-reporting (*n* = 35), and with over-reporting (*n* = 44) attitudes. The clinical record analysis focused on four types of information in which differences between these two subgroups emerged:

1. **Self-identification of problems:** leading to the clinical complaint: only three participants (9%) of the Under-Reporting group identified themselves as having personal difficulties/vulnerabilities on the basis of their complaints; the others identified difficulties associated with the context (i.e., physical health, professional and/or relational/family problems). On the contrary, in the Over-Reporting group, 44 participants (100%) identified personal difficulties/vulnerabilities, sometimes associated with physical health, professional and/or relational/family problems.
2. **Sick leave due to physical or mental health problems:** only four participants (11%) of the Under-Reporting group identified themselves as being on sick leave, not by mental problems, but by physical problems, while 20 participants (45%) of the Over-Reporting group stated being on sick leave, due to mental health problems.
3. **Types of clinical conditions identified by the psychologist:** performing the assessment: in the Under-Reporting group, the psychologists identified anxiety/depression in 10 participants (29%), behavioral problems (e.g., aggressive behavior, impulse control problems) in eight (23%), and alcohol and/or other substances problems in five participants (14%). In 12 cases (34%) the nature of the clinical problem was still unidentified. Pertaining to the Over-Reporting group, the psychologists identified anxiety/depression in 25 participants (57%), alcohol and/or other substance abuse problems in seven (16%), and adaptation/relational problems in four participants (9%). In eight cases (18%) the nature of the clinical problem was still unidentified.
4. **Self-reported history of psychological/psychiatric treatment and/or psychotropic medication:** only four participants (12%) of the Under-Reporting group described this kind of history (while this history was identified by the professional, in the clinical record, in the majority of the cases). On the contrary, in the Over-Reporting group, 36 (82%) participants reported history of clinical psychology intervention and/or psychiatry consultation and medication.

## Discussion and Conclusions

Our research aimed to investigate the differences between participants of two clinical samples, all of them outpatients with current professional activity, but some of them assessed in a traditional clinical context, while others assessed by a clinical psychologist of the professional organizations they work for. These comparisons focused a set of validity, clinical and personality psychopathology MMPI-2-RF scales. Our hypothesis was supported, as the Clinical-Organizational Sample has an under-reporting probability around five times higher than the one of participants assessed in a conventional clinical context T. Under-reporting is present in 15% of this sample, and defensiveness bias prevails, being present in 11% of the participants, while social desirability is less significant. This is in line with Keen et al. (2022) results, in which K-r showed incremental validity predicting lower scores on Higher-Order and Restructured Clinical scales. On the one hand, this may be due to the fact that the content of L-r scale items clearly identifies uncommon virtues, being more obvious and then easier to detect than the K-r scale items (Graham, Veltri, & Lee, 2022), that leading to a lower prevalence of Social Desirability, and consequently of lower Global under-reporting. On the other hand, the generalized perception that any kind of symptomology may hinder the individuals’ situation in the professional context, may explain this higher Defensiveness, since appearing well-adjusted may be more relevant than appearing especially virtuous. Nevertheless, it is pointed out that this would not be expected in a sample with clinical complaints. In the Clinical Sample, both types of under-reporting bias are residual, desirability being a little higher than defensiveness, as expected, since presenting psychological adjustment associated with symptom minimization (as this indicator suggests) is not expected in individuals with psychological suffering and seeking clinical help. This sample has displayed almost no under-reporting, and mainly an over-reporting response attitude was observed, as expected in clinical samples. The percentage was close to the 40% identified in the studies of Mittenberg et al. (2002), which included symptoms exaggeration, while most studies’ statistics consider simulation only. Thus, as the mean values of severe/rare complaints are at the lowest level of the over-reporting range (Ben-Porath & Tellegen, 2008/2011), these values may be regarded as a “cry for help”, as a strong need for recognition of perceived serious mental health problems, either conscious or unconscious, and that is a possible explanation for elevated responses on over-reporting scales (Young, 2019).

The Clinical-Organizational Sample presented higher scores on the under-reporting scales, and consequently, lower scores on most of the clinical and psychopathology scales, leading to lower clinical profiles. These results support the long-held clinical perspective, as well as results from empirical research, that motivations to under-reporting are associated with minimization of psychopathological symptoms (e.g., Bagby & Marshall, 2004).

When comparing the individuals with possible under-reporting attitude in the two samples, it is relevant that, in spite of the similarity in response attitudes, the Clinical-Organizational Sample has significantly lower values. These values may be considered excessively low since this sample is a clinical one, in which psychological symptomology is expected, with concomitant effect on the results of instruments like the MMPI-2-RF.

Pertaining to individuals with possible over-reporting in the two samples, there are no significant differences in the clinical scales, suggesting that, when it comes to emphasizing complaints and difficulties, either emotional, behavioral or thought related, that happens in a similar manner and with similar magnitude in different contexts.

In the Clinical-Organizational Sample, the response attitude bias, either under or over-reporting, has effect on most of the substantive scales, being more pronounced on scales more associated with claiming or denying emotional, cognitive, and somatic signs and symptoms, as well as internalizing conditions. Externalizing scales, and associated behavioral and relational characteristics, are the ones presenting less evidence or even absence of minimization or exacerbation of psychological difficulties. Probably, this happens because these scales do not reflect the personal suffering, either emotional or physical, which is more socially acceptable, but rather mainly impulsive and uncontrolled behavior (e.g., BXD, RC9 and ACT), with great impact on the professional context, work relations and organizational climate. In the same vein, other externalizing scales, pertaining to juvenile conduct problems or substance abuse, are not associated to over-reporting, probably because exacerbating this type of features would have an immediate negative impact on the professional context and on career expectations.

Among the personality psychopathology five measures, the scales identifying disconnection from reality (PSYC-r), anxiety, insecurity and worry (NEGE-r), and social disengagement and anhedonia (INTR-r) reflect the effect of response attitude bias, both under and over-reporting, probably because these scales are more related with emotional distress (Weiner & Greene, 2017). On the contrary, in the scales involving assertive and instrumental aggressive behavior (AGGR-r) and under-controlled, disconstrained or acting-out behavior (DISC-r), the three subsamples present similar scores, suggesting that these measures seem to be less changeable by clinical condition (Gómez et al., 2009), and eventually by response bias.

Further exploring the Clinical-Organizational Sample beyond the MMPI-2-RF results, several qualitative information, although from a subsample only, seems to be in line with the features characterizing under-reporting and over-reporting participants. The individuals with possible under-reporting do not identify psychological problems at the basis of their clinical complaints, but rather other problems, as physical health ones, while their psychologists do not corroborate this report. This may occur because identifying physical health problems is much less stigmatizing than assuming psychological difficulties or disorders, especially in the workplace. A systematic review identified that applicants with a mental health problem were rated as less employable than the ones with chronic physical disability (Brohan et al., 2012), and the same happens when applicants assume a history of past mental health *vs*. severe physical injury problems (Hipes et al., 2016). In the same line, the few individuals who report being on sick leave state that it is due to physical problems, and very few report past history of psychology/psychiatry and/or medication. On the other hand, pertaining to over-reporting, all the individuals with this response bias identified psychological problems on the basis of their complaints, almost half of them assumed being on sick leave due to mental health problems, and most of them reported history of clinical psychology and/or psychiatry treatment and medication.

Therefore, professionals should question the meaning of over-reporting in a Clinical-Organizational Sample. Within the framework of a motivational basis of response styles, the decision to exaggerate complaints can be viewed in terms of its predicted utility, based on both the available options and the desired outcome (Rogers, 2018). Therefore, over-reporting seems to be linked to specific motivations that may arise from professional circumstances, as a search for prolonged sick leaves, avoidance of certain professional tasks, an attempt to retire before legal age, or a plea for more help and support from the leadership. Thereby, it is of utmost relevance to thoroughly analyze each case where an over-reporting attitude is detected, when assessment takes place in a clinical-organizational context.

Thus, features of the professional context seem to affect the response attitude of clinical patients who are assessed and followed in this context, having an impact on the substantive scales’ scores. What seems to differentiate a Clinical-Organizational Sample from a common Clinical one is the Under-Reporting response attitude, as no mean level in the clinical and specific problems scales reaches T50, something completely uncommon in clinical patients, and not compatible with a psychopathological condition. In Rothermund et al. (2016) and Rothermund et al. (2017) studies, individuals receiving mental health consultation in the workplace presented lower symptom severity compared to individuals in the traditional clinical settings. As response attitudes were not assessed in these studies, we may speculate that underreporting could have been higher in the former patients, thus leading to lower clinical severity. Although the workplace has been acknowledged as a key context for early identification and treatment of mental health problems, and psychotherapeutic consultation has been considered similarly effective whether delivered in the workplace or in an outpatient clinic (Rothermund et al., 2016), our results highlight that response attitude should be considered. Ultimately, an under-reporting strategy on the MMPI may translate to an unwillingness to discuss problems in therapy (Graham, Veltri, & Lee, 2022), an attitude that may also affect the psychological intervention process.

Finally, the Clinical-Organizational context should not be addressed as a whole, but as two tendencies, when it comes to response distortion. The maximization of symptoms and problems is similar to the traditional clinical context, and may be understood in terms of stressing complaints in order to get a more immediate support. The minimization of problems and symptoms is prevalent, not consistent with the characterization of a clinical sample and it should be taken into account. Participants with over-reporting are individuals that emphasize general and specific complaints, reflecting on most of the scales. However, enhancing of emotional, thought and somatic, and cognitive difficulties, particularly physical distress, suicidal risk, and relational and social difficulties, stand out. There is no emphasis on behavioral problems. Participants with an under-report attitude almost totally deny emotional, aggressiveness and interpersonal relationship problems. Most of these individuals do not recognize a clinical history, and attribute their difficulties to physical, professional or relational/family problems. Literature shows that employees are afraid to disclosure mental health problems in the workplace because they fear stigma and discrimination by others, loss of credibility, as well as they fear the risk of job or financial loss (Brohan et al., 2012; Stratton et al., 2018). Despite confidentiality in the clinical consultation, defensiveness, as a partially non-conscious process (Graham et al., 2022) may still affect their attitude towards self-disclosure. Finally, considering the lowest levels of psychopathology in the clinical-organizational sample, it is possible that individuals with more severe psychopathology would have searched for psychological services outside their workplace. However, if this would be the case, this sample would just present low psychopathology levels, but not the higher mean under-reporting level it did. Finally, it is worth mentioning that many of the individuals of the clinical-organizational sample do not present under-report.

This study has some limitations. Although all participants are outpatients, there is some heterogeneity concerning disorders within each sample. In both samples, there is considerable prevalence of masculine participants, and that may result in a higher tendency to a biased response attitude (e.g., Dreber & Johannesson, 2008; Hogue et al., 2013). So, although this variable was controlled in the analyses, caution must be taken concerning generalization of the results to women, as they may present a different pattern. There are also differences in the number of individuals currently on sick leave between the samples, and, although that variable was also controlled, future studies should explore if difference in work situation may have an effect on response attitudes. Considering “real world” comparison groups were used, this study does not allow for stating of the *impact* of the context, as only an experimental design could reveal the impact of the assessment context in the assessment results. As strengths, however, to our best knowledge, this is the first study to explore response attitudes in the clinical-organizational context, and associated features of these patients, although assuming a correlational methodology. The patients’ response attitudes seem to have correlates with implications on clinical assessment, and consequently on clinical decision-making, pertaining to the need for clinical intervention with these patients.

At an applied level, relevance should be placed on the validity scales, and on the under and over-reporting indicators. In addition, to what extent response attitudes may have been influenced by contextual motives associated with the assessment request, and specific motivations to enhance or deny undesirable characteristics, and even psychopathology, should be taken into account. Considering clinical intervention in the workplace is progressively more relevant within the scope of mental health services, some precautions should be taken to maximize its efficacy and minimize potential hazards. Psychologists undertaking assessment and treatment in the clinical-organizational context should be aware of an enhanced predisposition for under-reporting. Otherwise, the risk of a biased diagnosis may be significant, allowing a professional with relevant psychological distress and maladjustment to continue his professional role, sometimes putting himself and other members of society at risk.

Ultimately, remaining doubts concerning response attitudes in the MPPI-2-RF, in each case, should be clarified with data from other instruments, or other assessment methods. In this regard, the consistency of data from different instruments, as well as its consistency with available clinical data, namely through clinical interview (i.e., behavioral observation, current ‘life’, and clinical history data) is of utmost importance.

It would be relevant to assess the differences between individuals with under-reporting and with over-reporting attitudes, in the Clinical-Organizational Sample. On the one hand, over-reporting is a call for attention to the symptoms, but on the other hand, it has also been related with premature termination of psychological treatment (Anestis et al., 2015), suggesting it may be more related to a tentative to be heard rather than a call for treatment, *per se* (Young, 2019).

## Data Accessibility Statement

The data that support the findings of this study are available on request from the corresponding author. The data are not publicly available due to privacy or ethical restrictions.
